# Can ^18^F-NaF PET/CT before Autologous Stem Cell Transplantation Predict Survival in Multiple Myeloma?

**DOI:** 10.3390/cancers12051335

**Published:** 2020-05-23

**Authors:** Christos Sachpekidis, Annette Kopp-Schneider, Maximilian Merz, Anna Jauch, Marc-Steffen Raab, Hartmut Goldschmidt, Antonia Dimitrakopoulou-Strauss

**Affiliations:** 1 Clinical Cooperation Unit Nuclear Medicine, German Cancer Research Center, 69120 Heidelberg, Germany; a.dimitrakopoulou-strauss@dkfz.de; 2 Department of Biostatistics, German Cancer Research Center (DKFZ), 69120 Heidelberg, Germany; kopp@dkfz-heidelberg.de; 3 Department of Internal Medicine V, University Hospital Heidelberg and National Center for Tumor Diseases (NCT), 69120 Heidelberg, Germany; maximilianmerz@web.de (M.M.); marc.raab@med.uni-heidelberg.de (M.-S.R.); hartmut.goldschmidt@med.uni-heidelberg.de (H.G.); 4 Institute for Human Genetics, University of Heidelberg, 69120 Heidelberg, Germany; anna.jauch@med.uni-heidelberg.de

**Keywords:** multiple myeloma, dynamic ^18^F-NaF PET/CT, two-tissue compartment model, standardized uptake value (SUV), progression-free survival (PFS)

## Abstract

There is an unmet need for positron emission tomography (PET) radiotracers that can image bone disease in multiple myeloma (MM) in a more sensitive and specific way than the widely used ^18^F-fluorodeoxyglucose (^18^F-FDG). Sodium fluoride (^18^F-NaF) is a highly sensitive tracer of bone reconstruction, evolving as an important imaging agent for the assessment of malignant bone diseases. We attempted to investigate for the first time the prognostic significance of ^18^F-NaF PET/CT in newly diagnosed, symptomatic MM patients planned for autologous stem cell transplantation (ASCT). Forty-seven patients underwent dynamic and static PET/CT with ^18^F-NaF before treatment. After correlation with the respective findings on CT and ^18^F-FDG PET/CT that served as reference, the ^18^F-NaF PET findings were compared with established factors of high-risk disease, like cytogenetic abnormalities as well as bone marrow plasma cell infiltration rate. Furthermore, the impact of ^18^F-NaF PET/CT on progression-free survival (PFS) was analyzed. Correlation analysis revealed a moderate, significant correlation of the ^18^F-NaF parameters SUV_average_ and K_1_ in reference tissue with bone marrow plasma cell infiltration rate. However, no significant correlation was observed regarding all other ^18^F-NaF PET parameters. Survival analysis revealed that patients with a pathologic ^18^F-NaF PET/CT have a shorter PFS (median = 36.2 months) than those with a physiologic scan (median = 55.6 months) (*p* = 0.02). Nevertheless, no quantitative ^18^F-NaF parameter could be shown to adversely affect PFS. In contrast, the respective analysis for quantitative dynamic ^18^F-FDG PET/CT revealed that the parameters SUV_max_, fractional blood volume (V_B_), k_3_ and influx from reference tissue as well as SUV_average_ from MM lesions had a significant negative impact on patient survival. The herein presented findings highlight the rather limited role of ^18^F-NaF PET/CT as a single PET approach in MM.

## 1. Introduction

Positron emission tomography/computed tomography (PET/CT) with the tracer ^18^F-fluorodeoxyglucose (^18^F-FDG) is a powerful diagnostic tool in multiple myeloma (MM) for the detection of medullary and extramedullary disease (EMD), a reliable predictor of survival as well as the modality for treatment response evaluation [1,2,3,4,5]. According to the latest update of the International Myeloma Working Group (IMWG), the detection of one or more osteolytic lesions on CT or PET/CT fulfills the criteria of bone disease in patients with bone marrow plasma cell infiltration of ≥10% or histologically proven plasmocytoma and, therefore, of symptomatic MM requiring treatment [5].

However, ^18^F-FDG PET/CT carries some limitations. Firstly, its sensitivity in particular for detection of diffuse bone marrow infiltration and skull lesions is relatively poor, with the incidence of PET false-negativity reaching 11%, a finding attributed mainly to the significantly lower expression of the gene coding for hexokinase-2, which catalyzes the first step of glycolysis [6]. Moreover, ^18^F-FDG, as a glucose analog, is generally restricted in oncological imaging by both false-positive (inflammation, post-surgical areas, metallic implants, recent use of chemotherapy, fractures, etc.) and false-negative results (hyperglycemia, recent administration of high-dose steroids, etc.) [7,8]. Further, the lack of widely applied criteria for image interpretation of ^18^F-FDG PET/CT in MM leads to a rather poor interobserver reproducibility in scan interpretation, a recently addressed issue [9]. These limitations of ^18^F-FDG as a biomarker of MM render the development of other more sensitive and specific PET tracers, potentially targeting different molecular pathways, necessary.

Sodium fluoride (^18^F-NaF) is another PET tracer used for skeletal imaging. The bone uptake of ^18^F-NaF reflects regional blood flow, osteoblastic activity and bone turnover [10,11,12]. ^18^F-NaF PET/CT is evolving as a highly sensitive imaging modality of bone reconstruction, with potential indications in a wide range of bone disease [13,14,15]. Particularly in MM, the use of the radiopharmaceutical can be theoretically justified by the fact that bone disease is a major cause of morbidity and mortality, and that practically all MM patients develop bone involvement during the course of the disease [5]. Indeed, ^18^F-NaF PET/CT has been applied in several studies of MM with results ranging from rather discouraging [16,17,18,19] to promising [20,21,22,23]. In this context, the role of ^18^F-NaF in MM is yet to be clarified.

With the current prospective study, we investigated for the first time the prognostic significance of qualitative and quantitative parameters derived from dynamic and static ^18^F-NaF PET/CT in MM. We compared findings from ^18^F-NaF PET/CT with established factors of high-risk disease, like cytogenetic abnormalities, as well as bone marrow plasma cell infiltration rate, and analyzed the impact on progression-free survival (PFS) after autologous stem cell transplantation (ASCT) in patients with newly diagnosed, symptomatic MM. A comparison with respective findings derived from ^18^F-FDG PET/CT examinations, performed one day before, in the same cohort was also made.

## 2. Materials and Methods

### 2.1. Patients

Forty-seven patients (31 male, 16 female; median age 59.9 years) with previously untreated, symptomatic MM based on the criteria established by the IMWG (2003) were included in the study [24]. All patients underwent high-dose chemotherapy (HDT) followed by ASCT. Twenty-two patients (45.8%) were enrolled in the prospective GMMG MM5 phase III trial that compared two different bortezomib-based induction therapies, followed by ASCT and lenalidomide consolidation as well as maintenance therapy for two years or until complete response [25]. Twenty-five patients (53.2%) were treated outside the MM5 trial with comparable treatment regimens and ASCT. The same cohort has been evaluated in a previous publication of our group in a different analysis with regard to ^18^F-FDG PET/CT [26]. Neither diabetic patients nor patients under steroid therapy at the time of PET/CT scanning were included in our analysis. Patients gave written informed consent after the study was fully explained to them. Our study was conducted in accordance to the declaration of Helsinki, and was approved by the ethical committee of the University of Heidelberg (S-076/2010) and the Federal Agency of Radiation Protection in Germany (“Bundesamt für Strahlenschutz”).

### 2.2. PET/CT Data Acquisition

All patients underwent ^18^F-NaF PET/CT. Data acquisition consisted of two parts: the dynamic part (dPET/CT studies) and the static part (whole-body PET/CT). dPET/CT studies were performed over the lower abdomen and the pelvic area after intravenous administration of maximum 250 MBq ^18^F-NaF for 60 min using a 24-frame protocol (10 frames of 30 s, 5 frames of 60 s, 5 frames of 120 s and 4 frames of 600 s). Whole-body, static imaging was performed in all patients with an image duration of 2 min per bed position for the emission scans after the end of the dynamic acquisition. A dedicated PET/CT system (Biograph mCT, S128, Siemens Co., Erlangen, Germany) with an axial field of view of 21.6 cm with TruePoint and TrueV, operated in a three-dimensional mode was used. The system allows the simultaneous acquisition of 56 transversal slices (1 bed position) with a theoretical slice thickness of 2.036 × 2.036 × 4 mm [27]. A low-dose attenuation CT (120 kV, 30 mA) was used for attenuation correction of the dynamic emission PET data and for image fusion. A second low-dose CT (120 kV, 30 mA) was performed after the end of the dynamic series covering the area from the skull to the feet. All PET images were attenuation corrected and an image matrix of 400 × 400 pixels was used for iterative image reconstruction. Iterative image reconstruction was based on the ordered subset expectation maximization algorithm (OSEM) with 2 iterations and 21 subsets as well as time of flight (TOF).

### 2.3. PET/CT Data Analysis

Data analysis consisted of qualitative analysis of the PET/CT scans, semi-quantitative evaluation based on standardized uptake value (SUV) calculations, and quantitative analysis of the dynamic ^18^F-NaF PET data. 

Qualitative analysis was based on visual assessment of the PET/CT scans. Skeletal foci of enhanced ^18^F-NaF uptake were classified as MM, based on the results of the underlying low-dose CT and the ^18^F-FDG PET/CT performed one day before, which served as reference. Briefly, only those ^18^F-NaF positive skeletal lesions that corresponded to lytic lesions on CT or to respective ^18^F-FDG-positive skeletal lesions, for which another benign (e.g., degenerative, posttraumatic) etiology was excluded, were considered suggestive of MM [16,18,19].

Semi-quantitative evaluation was based on volumes of interest (VOIs) and on subsequent calculation of SUV values. VOIs were drawn with an isocontour mode (pseudo-snake) [28] and were placed over reference tissue as well as over the hottest, focal MM lesion in each patient, if present. The iliac bone, if without focal tracer enhancement, served as reference tissue. In cases of focal lesions in the iliac bone, the same bone of the opposite side served this purpose.

Quantitative evaluation of the dynamic ^18^F-NaF PET/CT data of the lower abdomen and the pelvic area was also performed in the hottest MM lesion and in reference tissue (iliac bone). The analysis was performed using a dedicated software (PMOD Technologies Ltd., Zürich, Switzerland) and based on a two-tissue compartment model with a blood component (fractional blood volume, V_B_) with methods already reported in literature and performed previously by our group [16,18,19,29,30,31]. The input function was retrieved from PET/CT data. Although the accurate measurement of the input function requires arterial blood sampling, it can be retrieved relatively simplified and non-invasively from the image data with good accuracy [32]. In particular, the mean value of the VOI data from a large arterial vessel of the lower abdomen/pelvic area (descending aorta or common iliac artery) was used. A vessel VOI consisted of at least seven ROIs in sequential PET/CT images. The recovery coefficient was 0.85 for a vessel diameter of 8 mm. For the input function, we used vessels with no signs of atherosclerotic plaques in CT for the measurements. A limitation of compartment modeling is that the assessment of the transport rates can be operator dependent. The reason is that these models use an iterative fitting to calculate the least squares between measured and model data, which may lead to overfitting problems. Noise in the time–activity curves (TACs) and, in particular, inappropriate input (TACs) have an impact on the assessed rates. A robust solution to overcome these problems has been published by a group, based on machine learning approaches and a large oncological reference database [31,33].

Briefly, in the case of ^18^F-NaF, the two-tissue compartment model consists of the vascular compartment (plasma), the extravascular bone compartment, and the bone mineral compartment. Application of this model leads to the extraction of the kinetic parameters K_1_, k_2_, k_3_ and k_4_ as well as influx (K_i_). The rate constants K_1_ and k_2_ describe fluoride ion clearance from plasma to the extravascular compartment, reflecting the exchange of the ions between plasma and bone interstitial fluid. The parameters k_3_ and k_4_ describe the exchange of fluoride with hydroxyl groups of hydroxyapatite crystal of the bone, representing the formation of fluorapatite and the opposite. Influx (K_i_) is derived from the equation = (K_1_ × k_3_)/(k _2_ + k_3_) and reflects the net clearance of ^18^F-NaF to the bone mineral compartment and, presumably, represents the bone remodeling rate [12]. V_B_ reflects the fraction of blood within the VOI. 

In addition to compartment modeling, fractal analysis, a non-compartment model, was used in order to calculate the parameter of heterogeneity and complexity, expressed by a non-integer value, so-called fractal dimension (FD). FD is calculated for the time–activity data in each individual voxel of a VOI and represents tissue heterogeneity based on the box counting procedure of chaos theory. The values of FD vary from 0 to 2, showing the more deterministic or chaotic distribution of the tracer activity respectively [34].

### 2.4. Clinical Parameters, Bone Marrow Plasma Cell Infiltration and Fluorescence In Situ Hybridization 

All patients included in the study received bone marrow aspirates or biopsies performed within four weeks around the ^18^F-NaF PET/CT examination. Percentage of bone marrow infiltration by malignant plasma cells was assessed via light microscope from Giemsa-stained bone-marrow smears [35]. The infiltration rate represents the number of plasma cell in comparison to all nucleated, hematopoietic cells in the bone marrow. For the definition of high-risk disease, the Revised International Staging System (R-ISS) score was defined before start of therapy. Based on this prognostic system, stage R-ISS I included patients of ISS stage I (serum β2-microglobulin level < 3.5 mg/L and serum albumin level ≥ 3.5 g/dL), no high-risk chromosomal abnormalities, and normal lactate dehydrogenase (LDH) level; stage R-ISS III included ISS stage III (serum β2-microglobulin level > 5.5 mg/L) and high-risk chromosomal abnormalities [del(17p) and/or t(4;14) and/or t(14;16)] or high LDH level; stage R-ISS II included all the other possible combinations [36]. Fluorescence in situ hybridization (FISH) was performed, as described previously [37], on CD138-purified plasma cells using the following probes: 1q21, 5p15, 5q35, 8p21, 9q34, 11q22.3, 13q14, 15q22, 17p13, and 19q13. We also investigated immunoglobulin H (IgH) translocations using an IgH break-apart probe as well as probes for t(11;14), t(4;14) and t(14;16).

### 2.5. Statistical Analysis

Spearman correlation analysis was performed to investigate the correlation between quantitative PET parameters, since some of these parameters showed a skewed distribution. PFS was measured from the date of start of treatment until disease progression or death from any cause. Kaplan–Meier estimates were generated and median PFS estimated. Median follow-up time was determined by inverse Kaplan–Meier estimation. For univariate comparison of PFS, a log-rank test was used. Multivariate Cox proportional hazards regression analysis for all ^18^F-NaF PET parameters adjusting for different risk groups determined at start of therapy was applied. Maximally selected rank statistics were used to identify the optimal cut point of quantitative covariables used for dichotomization of the cohort with respect to PFS. Statistical analysis was performed using R version 3.6.1 (The R Foundation for Statistical Computing 2019) and R packages survival and maxstat. The results were considered significant for *p* values less than 0.05 (*p* < 0.05). 

## 3. Results

### 3.1. Patient Cohort

The plasma cell infiltration, as derived from bone marrow aspirates or biopsies from the iliac crest, ranged between 1% and 92%, with a mean value of 40% (median = 32%). Cytogenetic data were available in 40 patients (85%), with high-risk cytogenetic abnormalities being detected in 8/40 (20%) of them. A combination of the ISS and cytogenetic data was available in 36 patients. Based on this, 14 patients were classified in the R-ISS-1 group (38.9%), 20 patients in the R-ISS-2 group (55.5%), and two patients in the R-ISS-3 group (5.6%) (Table 1).

### 3.2. ^18^F-NaF PET/CT Findings

**Figure 1:**
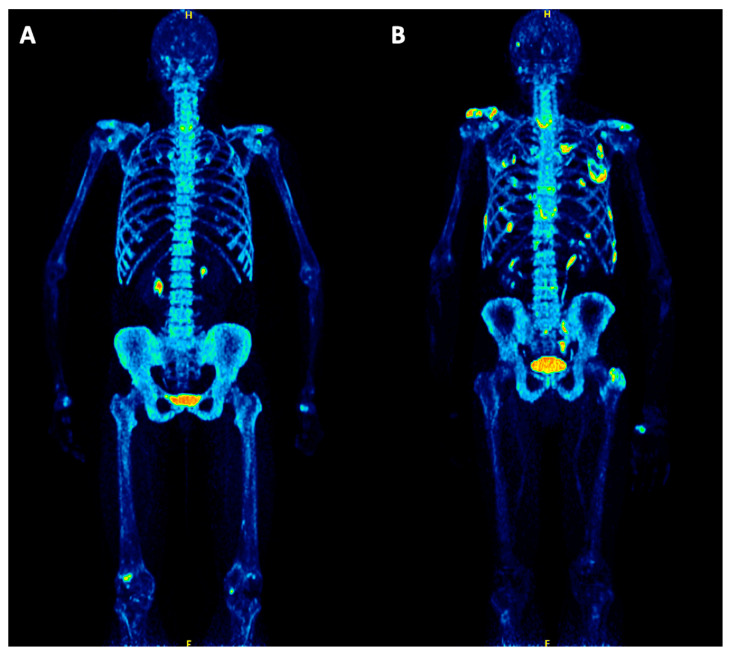
Maximum intensity projection (MIP) PET/CT images of two symptomatic multiple myeloma (MM) patients before treatment. (**A**) PET/CT of a 66-year-old female patient shows no ^18^F-NaF positive, skeletal myeloma lesions. However, several degenerative changes are depicted, for example in the spine, shoulders, hands and knees. (**B**) PET/CT of a 60-year-old male patient demonstrating multiple focal, ^18^F-NaF positive myeloma lesions in the scapula, humerus, spine, pelvis, femur and ribs, partially corresponding to pathologic rib fractures.

Twenty-seven patients (57%) had a pathologic ^18^F-NaF PET/CT, while the remaining 20 patients (43%) had no pathologic findings on PET/CT (Figure 1). No EMD was detected with this tracer in the cohort. Patients with a pathologic ^18^F-NaF pattern had a median bone marrow plasma cell infiltration of 41.0% (mean = 45.7), compared to a median of 19.5% (mean = 30.5%) of those with no pathologic findings on PET/CT (*p* = 0.07). Regarding relation between PET distribution and results of cytogenetic analysis it was found that 5/8 patients (62.5%) with high-risk abnormalities demonstrated a pathologic PET pattern, while 3/8 patients (37.5%) were PET negative. Respectively, 19/32 (59%) patients with standard cytogenetic risk had a pathologic PET, while 13 of them (41%) were PET negative.

As mentioned above, semi-quantitative (based on SUV calculations) and quantitative analyses of the ^18^F-NaF PET data (based on two-tissue compartment modeling and fractal analysis) were performed both in reference tissue (os ilium) and in the hottest focal MM lesion in each patient with such lesions. The descriptive statistics of these calculations are presented in Table 2. No statistically significant differences were observed between patients of different ISS and R-ISS groups regarding any ^18^F-NaF PET parameter.

### 3.3. Correlation between ^18^F-NaF PET/CT Parameters, Bone Marrow Plasma Cell Infiltration and Cytogenetics

Exploratory correlation analysis between the quantitative PET parameters and bone marrow plasma cell infiltration revealed that in reference tissue, SUV_average_ and K_1_ correlated moderately but significantly with bone marrow infiltration by malignant plasma cells (r = 0.30 and r = 0.35, respectively). In contrast, no significant correlation was observed with all other parameters from reference tissue as well as those from MM lesions. Moreover, no statistically significant differences were observed between patients with high-risk abnormalities and those with standard cytogenetic risk regarding any PET parameter both in reference tissue and MM lesions, with *p* values ranging between 0.14 (SUV_max_ of reference tissue) and 0.94 (K_1_ of MM lesions).

### 3.4. Correlation between ^18^F-NaF PET/CT and PFS

**Figure 2:**
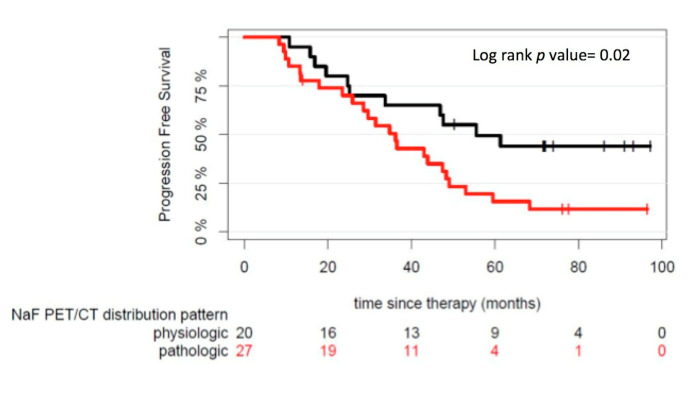
Progression-free survival (PFS) outcome after start of therapy according to physiologic and pathologic ^18^F-NaF PET/CT distribution patterns. The numbers of patients at risk in each group and for the respective time-points are shown below the plots.

Based on the visual/qualitative evaluation of the whole-body PET/CT scans, we found that patients with a pathologic ^18^F-NaF PET/CT demonstrated a median PFS of 36.2 months, compared to 55.6 months in the group of patients with a physiologic (negative) ^18^F-NaF distribution pattern (*p* = 0.02) (Figure 2).

Quantitative PET parameters in reference tissue and MM lesions were dichotomized at the median to investigate their effect on PFS. However, no statistically significant differences in PFS were observed between patients with parameters below the median versus patients with values above the median. Moreover, no optimal cut-offs for these parameters regarding PFS prediction could be identified using maximally selected rank statistics. Furthermore, multivariable Cox proportional hazards regression analysis for all ^18^F-NaF PET parameters adjusting for different risk groups was also performed and revealed no significant impact on PFS.

**Figure 3:**
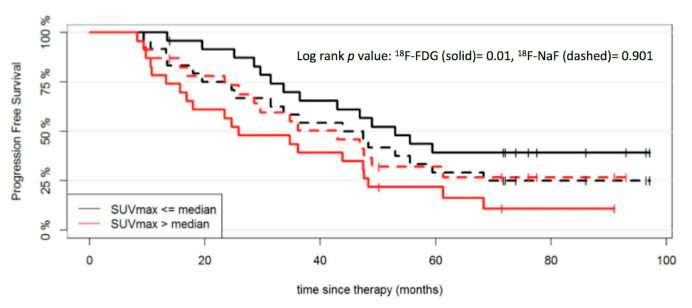
PFS outcome after start of therapy according to SUV_max_ derived from reference tissue for ^18^F-NaF and ^18^F-FDG PET/CT. The patient cohort is dichotomized at the median SUV_max_ values for ^18^F-NaF (12.9) and ^18^F-FDG (3.3).

**Figure 4:**
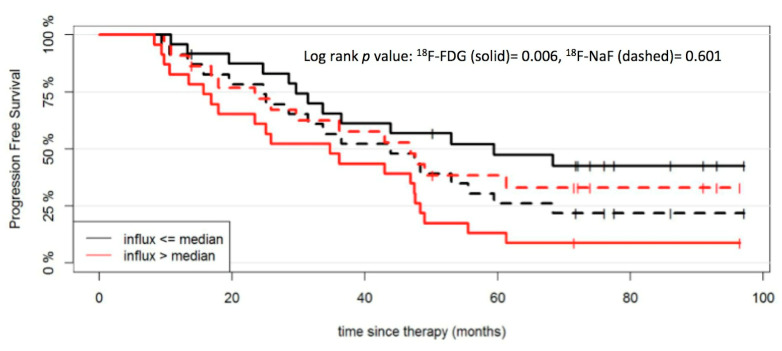
PFS outcome after start of therapy according to influx derived from reference tissue for ^18^F-NaF and ^18^F-FDG PET/CT. The patient cohort is dichotomized at the median influx values for ^18^F-NaF (0.07 mL min^−1^ mL^−1^) and ^18^F-FDG (0.01 mL min^−1^ mL^−1^).

A survival analysis was also performed for quantitative, dynamic ^18^F-FDG PET/CT. Similarly to ^18^F-NaF PET, quantitative ^18^F-FDG PET parameters in reference tissue and MM lesions were also dichotomized at the median to investigate their effect on PFS. According to this, the PFS of patients with parameters SUV_max_ (*p* = 0.01), V_B_ (*p* = 0.02), k_3_ (*p* = 0.04) and influx (*p* = 0.006) from reference tissue as well as SUV_average_ from MM lesions (*p* = 0.02) above the median value was significantly shorter compared to patients with respective values below the median (Figure 3 and Figure 4). The respective descriptive statistics for ^18^F-FDG PET parameters are presented in a previous publication of our group [26].

## 4. Discussion

The determination of the extent of bone disease is one of the most challenging tasks in MM diagnostics, with implications in patient quality of life and prognosis. In this context, the role of PET/CT with the tracer ^18^F-FDG has been highly explored and upgraded, as reflected by the increasing amount of literature published in the field [1,2,3,4,5]. However, despite its nowadays recognized role in MM diagnosis, prognosis and treatment response evaluation, ^18^F-FDG PET/CT is far from being considered an optimal biomarker for the disease, due to its several limitations.

In an attempt to overcome these limitations of ^18^F-FDG as an imaging biomarker of MM, several other PET tracers have been proposed and tested in smaller patient studies with this malignancy. For example, ^11^C-methionine, an aminoacidic PET tracer mainly employed in the diagnosis of central nervous system (CNS) tumors, has been shown to be superior over ^18^F-FDG for staging and re-staging of both intra- and extramedullary MM lesions [38,39,40], and can moreover highlight more rare and radiologically challenging sites of EMD such as the CNS [41]. ^68^Ga-pentixafor, a radiolabeled peptide with high affinity for chemokine receptor 4 (CXCR4), which is involved in myeloma cell homing, bone marrow retention, angiogenesis and metastasis, has shown very promising results regarding both its diagnostic performance [42,43] and its potential application as a theranostic agent, identifying candidates suitable for CXCR4-directed therapies with β-emitting radionuclides such as lutetium-177 (^177^Lu). The first results of this approach have demonstrated that this form of ^68^Ga-pentixafor-tailored endoradiotherapy is feasible and successful even at an advanced MM stage [44]. However, further studies in large patient cohorts are needed to assess the clinical potential of these tracers within the MM work-up.

^18^F-NaF is a very sensitive and reproducible PET tracer for the quantification of bone metabolism [10,11,12]. In recent years, several studies have investigated the potential role of ^18^F-NaF PET/CT in detection of MM bone lesions with rather controversial results [16,17,18,19,20,21,22,23]. At present, PET/CT with ^18^F-NaF, similar to all non-^18^F-FDG tracers, is not part of the diagnostic algorithm of MM.

Our work aimed at comparing findings of ^18^F-NaF PET/CT with recognized biomarkers of MM as well as investigating the prognostic significance of this imaging modality in the disease. Due to the known high incidence of false-positive findings on ^18^F-NaF, as a highly sensitive imaging agent of bone reconstruction, the characterization of a ^18^F-NaF PET scan as pathologic was based on the presence of focal, ^18^F-NaF avid lesions only after comparison with the underlying low-dose CT and the ^18^F-FDG PET/CT performed one day before, which are recognized biomarkers of bone disease in MM and thus served as reference. Firstly, a comparison of the ^18^F-NaF PET/CT findings with bone marrow plasma cell infiltration was performed. We found that patients with a pathologic ^18^F-NaF PET/CT had a non-significantly higher bone marrow plasma cell infiltration rate than those with a normal PET/CT scan. Correlation analysis revealed that in reference skeleton, the intensity of tracer uptake, reflected by SUV_average_, and fluoride ion clearance from plasma to the extravascular compartment, reflected by K_1_, correlated moderately but significantly with bone marrow infiltration by malignant plasma cells. Similar results were obtained in a study by our group comparing the tracer ^18^F-FDG with results of bone marrow infiltration [34]. That study had also documented a moderate, significant correlation of the parameters SUV_average_ and K_1_ with the rate of infiltration by malignant plasma cells in reference tissue (bone marrow of the iliac bone). However, significant correlations were also observed with other quantitative ^18^F-FDG PET parameters such as SUV_max_, influx (K_i_) and FD, which was not the case in the present study. 

We further compared ^18^F-NaF PET/CT findings with the results of cytogenetic analysis. A total of 62.5% of the patients with high-risk abnormalities had a pathologic PET scan and 37.5% of them were PET negative, with no significant differences between patients with high-risk abnormalities and those with standard cytogenetic risk. 

EMD is another feature recognized to adversely affect survival in MM [1,45,46]. Despite the presence of four patients with known EMD in this cohort based on ^18^F-FDG PET/CT [26], none could be identified by means of ^18^F-NaF PET/CT, highlighting a disadvantage of the tracer.

The main focus of the present study was the investigation of the potentially predictive role of ^18^F-NaF PET/CT on MM survival. Survival analysis revealed that a pathologic ^18^F-NaF PET/CT scan is associated with a statistically significantly shorter PFS than a normal one. However, no association was observed between any ^18^F-NaF parameter, derived either from reference tissue or MM lesions and PFS. In comparison, the respective analysis in the same patient cohort with the tracer ^18^F-FDG revealed a significant adverse effect of the parameters SUV_max_, V_B_, k_3_ and influx from reference tissue as well as SUV_average_ from MM lesions on patient survival. These findings on ^18^F-FDG PET/CT are actually the outcome of an updated survival analysis in the same patient cohort previously studied and published by our group in 2019 [26]. 

These findings are not surprising regarding the performance of ^18^F-NaF PET/CT in MM. Previous studies from our and other groups had shown that the detection rate of MM bone lesions by ^18^F-NaF PET imaging was lower than that of ^18^F-FDG PET, while specificity issues were also raised due to the nature of the tracer [16,17,47]. In particular, in a study of 60 MM patients examined with both ^18^F-FDG PET/CT and ^18^F-NaF PET/CT, the latter could detect only 39% of the myeloma lesions detected by ^18^F-FDG PET/CT [16]. In line with this, Ak et al. showed that ^18^F-NaF PET/CT detected 57 of the 128 MM lesions demonstrated on ^18^F-FDG PET/CT in a group of 26 patients. In addition, 135 bone lesions attributed to fractures and degenerative changes were depicted with ^18^F-NaF PET/CT, stressing the low specificity of the tracer [17]. Further, in a cohort of 30 patients (10 each with monoclonal gammopathy of unknown significance (MGUS), smoldering multiple myeloma (SMM) and MM) evaluated with conventional skeletal surveys, ^18^F-FDG PET/CT, ^18^F-NaF PET/CT, and dynamic contrast enhanced MRI (DCE-MRI), ^18^F-NaF PET provided no additional information in addition to that already present on the CT component [47]. The explanation for the low sensitivity of ^18^F-NaF PET in detection of MM bone disease can be found in the nature of both the myeloma lesions and the radiotracer: myeloma bone disease is purely osteolytic with absent or minimal osteoblastic activity [48]. However, the uptake of ^18^F-NaF reflects osteoblastic activity, resulting in lack of depiction of several lytic lesions on PET imaging. The same mechanism can also explain the limited specificity of ^18^F-NaF PET. Any cause of bone reconstruction and/or newly mineralizing bone, irrelevant of its etiology, leads to increased accumulation of ^18^F-NaF, resulting in a high incidence of false-positive findings, such as in traumatic or degenerative bone lesions [16,18].

Interestingly, some recent studies have assessed the role of ^18^F-NaF PET/CT in treatment response assessment of MM with controversial results. Wang et al. studied with ^18^F-NaF PET/CT six patients from a phase I clinical trial of a monoclonal antibody targeting the glycoprotein DKK1 (Dickkopf-1) before study entry and after completing six cycles of therapy. The changes in SUV_average_ in the lumbar spine and the left hip were calculated, revealing a statistically significant increase as response to treatment [21]. In contrast, Nakuz et al. reported on a statistically significant SUV_max_ decrease in MM lesions in seven patients examined with ^18^F-NaF PET/CT before and after treatment. All patients of that cohort showed either a very good partial response or complete response according to the IMWG guidelines. However, they were treated with different anticancer regimens, including radiotherapy, immunochemotherapy and ASCT, and had varying times of follow-up scans [49]. A significant drawback of both studies is the very low number of patients included. In the largest cohort of MM patients (*n* = 29) studied with both ^18^F-FDG PET/CT and ^18^F-NaF PET/CT before and after HDT/ASCT, ^18^F-NaF yielded rather disappointing results both in initial evaluation of disease extent and in the assessment of treatment response. Baseline ^18^F-NaF PET/CT was negative in 14% of the ^18^F-FDG PET/CT-positive MM patients, in total depicting only 43% of the ^18^F-FDG-positive lesions. Regarding follow-up studies, ^18^F-NaF PET/CT showed persistence of the majority (82%) of the baseline ^18^F-NaF positive MM lesions after treatment, despite the fact that 65% of them had turned ^18^F-FDG negative as response to treatment. It is of note that in that study, SUV values as well as the kinetic parameters K_1_ and influx from reference skeleton demonstrated a significant decrease after therapy, despite the persistence of most focal MM lesions [19]. Most recently, Zadeh et al. followed, with ^18^F-NaF PET/CT, two groups of MM patients undergoing different therapeutic regimens: the first received HDT/ASCT (*n* = 19 patients), and the second received conventional chemotherapy (*n* = 11 patients). The authors applied a CT-based algorithm to segment the bones, and the global mean SUV of the whole bone, pelvis and femoral neck was calculated. The main finding of this study was that the global ^18^F-NaF uptake significantly decreased after treatment in the group treated with HDT/ASCT, but not in the group receiving conventional chemotherapy. However, the two patient groups had different follow-up periods (8 weeks vs. 2 weeks), rendering direct comparison between them difficult [50]. The results of the aforementioned studies do not allow for any robust conclusions to be drawn regarding the potential role of ^18^F-NaF PET/CT in treatment assessment of MM.

The major limitation of the current study is the relatively small number of patients studied. This can be, however, justified, given the longer acquisition time of dynamic PET scanning, which is a necessity if quantitative/kinetic data are to be acquired. Other limitations include the lack of histological confirmation of the ^18^F-NaF avid focal lesions as well as the fact that dynamic sequences were performed only in the lower abdomen and the pelvis. The recent advent of new PET/CT scanners, which allow dynamic studies over several bed positions by using a continuous movement, will facilitate the use of dynamic PET protocols and reduce the acquisition time [51]. Finally, we focused on the predictive value of baseline imaging findings and patient characteristics before start of treatment, without performing further analyses including response to the applied treatment, e.g., induction therapy, ASCT or even best response after initial imaging, as it was not topic of this study.

## 5. Conclusions

In an attempt to investigate for the first time the prognostic significance of ^18^F-NaF PET/CT in patients with newly diagnosed, symptomatic MM, we compared findings derived from dynamic and static PET/CT with established factors of high-risk disease, including bone marrow plasma cell infiltration rate and cytogenetic abnormalities, and analyzed the impact on PFS. Apart from a moderate, significant correlation of SUV_average_ and K_1_ in reference tissue with bone marrow plasma cell infiltration rate, no other significant correlation was observed regarding the rest of the ^18^F-NaF PET parameters. Survival analysis revealed that patients with a pathologic ^18^F-NaF PET/CT have a shorter PFS than those with a physiologic scan. However, no quantitative ^18^F-NaF PET parameter affected PFS. In contrast, the quantitative parameters SUV_max_, V_B_, k_3_ and influx from reference tissue as well as SUV_average_ from MM lesions derived from ^18^F-FDG PET/CT adversely affect PFS. Although further studies are required, the herein presented findings highlight the rather limited role of ^18^F-NaF PET/CT, evaluated by means of quantitative analysis based on SUV calculations and two-tissue compartment modeling, in MM survival prognosis. Based on these and previously published results, the application of ^18^F-NaF PET/CT as a single PET approach in MM is not recommended. It should be noted, however, that ^18^F-NaF can provide essential information on bone remodeling and the patient’s skeletal history, and thus it could be performed in addition to validated imaging techniques, according to clinical indication.

## Figures and Tables

**Table 1:** Baseline patient characteristics.

Baseline Characteristics	*n*
Median age, years	59.9 [38.4–73.5]
Gender	
Male	31 (66%)
Female	16 (34%)
Median albumin, g/dL	4.2 [0.5–5.0]
Median β2-microglobulin, mg/L	2.8 [1.1–37.0]
Median LDH, u/L	184.0 [117.0–283.0]
Median bone marrow plasma cell infiltration	32% [1–92%]
Cytogenetic abnormalities	
High-risk [del(17p) and/or t(4;14) and/or t(14;16)]	8 (20.0%)
Standard risk	32 (80.0%)
ISS	
1	26 (61.9%)
2	12 (28.6%)
3	4 (9.5%)
R-ISS	
1	14 (38.9%)
2	20 (55.6%)
3	2 (5.6%)

Figures in parentheses are percentages; figures in brackets are ranges. LDH, lactate dehydrogenase.

**Table 2:** Descriptive statistics of SUV and kinetic parameters for ^18^F-NaF in reference bone and the hottest MM lesions. K_1_ and influx (K_i_) are expressed in ml min^−1^ mL^−1^. k_3_ is expressed in min^−1^. SUV values and FD have no unit. Blood volume (V_B_), as a fraction, also has no unit.

Parameters	Reference Tissue (os ilium)				MM Lesions			
	Median	Mean	SD	*n*	Median	Mean	SD	*n*
**SUV_average_**	8.1	8.2	2.5	47	14.4	20.1	15.8	14
**SUV_max_**	12.9	13.5	4.5	47	24.4	35.2	28.9	14
**V_B_**	0.004	0.03	0.08	47	0.003	0.03	0.04	10
**K_1_**	0.25	0.27	0.11	47	0.24	0.30	0.16	10
**k_3_**	0.05	0.05	0.15	47	0.24	0.27	0.19	10
**Influx (K_i_)**	0.07	0.07	0.03	45	0.08	0.13	0.09	10
**FD**	1.38	1.37	0.05	47	1.40	1.38	0.08	10

SD, standard deviation; *n*, number of evaluated lesions; SUV, standardized uptake value; FD, fractal dimension.
